# Behavioral Assessment of Unilateral Spatial Neglect with the Catherine Bergego Scale (CBS) Using the Kessler Foundation Neglect Assessment Process (KF-NAP) in Patients with Subacute Stroke during Rehabilitation in Japan

**DOI:** 10.1155/2021/8825192

**Published:** 2021-02-09

**Authors:** Daisuke Nishida, Katsuhiro Mizuno, Masatoshi Tahara, Shiori Shindo, Yuki Watanabe, Hiroki Ebata, Tetsuya Tsuji

**Affiliations:** ^1^Department of Physical Rehabilitation, National Center Hospital, National Center of Neurology and Psychiatry, 4-1-1 Ogawa-higashi-cho Kodaira, Tokyo 187-8551, Japan; ^2^Department of Rehabilitation Medicine, Keio University School of Medicine, 35 Shinanomachi Shinjuku, Tokyo 160-8582, Japan; ^3^Saiseikai Higashi-Kanagawa Rehabilitation Hospital, 1-13-10 Nishi-kanagawa, Kanagawa-ward Yokohama, Kanagawa 221-0822, Japan

## Abstract

The Kessler Foundation Neglect Assessment Process (KF-NAP) is an assessment tool for unilateral spatial neglect (USN), which is the scoring method for the Catherine Bergego Scale (CBS) based on detailed instructions. This study is aimed at determining the reliability and validity of the Japanese version of the KF-NAP (KF-NAP-J), evaluating the improvement of neglect assessment with KF-NAP-J, and comparing it with the original CBS for subacute stroke patients. We assessed subacute stroke patients admitted to our intensive rehabilitation hospital. Two KF-NAP-trained occupational therapists (OTs) assessed 22 patients. Before implementing the KF-NAP at the hospital, two other OTs assessed the other 23 patients using the CBS. We evaluated the interrater reliability of the KF-NAP and CBS using intraclass correlation coefficients (ICC) for the total scores, weighted kappa statistics for each subscale, and internal consistency using Cronbach's alpha. We assessed the validity of the KF-NAP against the Behavioral Inattention Test (BIT) and Functional Independence Measure (FIM) using Spearman's correlation coefficient. The reliability of both the KF-NAP and CBS was excellent. The weighted kappa results demonstrated that each subscale was in better agreement with the KF-NAP than with the CBS. In the KF-NAP, all eight subscales in which weighted kappa could be calculated were in significant agreement, and two were almost in perfect agreement. The KF-NAP moderately correlated with the subscales of BIT and FIM representing USN and activities of daily living. The USN detection rates of KF-NAP and BIT in the KF-NAP group were 63.6% and 22.7%, respectively. These results suggest that the KF-NAP, as well as the CBS, is useful to assess USN, which strongly impacts the rehabilitation outcomes in subacute stroke patients.

## 1. Introduction

Unilateral spatial neglect (USN) is defined as a failure to report, respond, or orient to novel or meaningful stimuli presented to the side opposite a brain lesion, when this failure cannot be attributed to either sensory or motor defects [1]. This disorder can be challenging for recovery and affect rehabilitation outcomes with respect to the activities of daily living (ADLs) [2, 3, 4, 5, 6]. Paper-and-pencil tests, such as the Albert test, Bells test, and Behavioral Inattention Test (BIT) [7], are widely used in clinical practice for the screening of USN. However, they have limitations in evaluating the effects of spatial neglect on the ADLs and may underdiagnose auditory and/or proprioceptive spatial neglect [8]. The Catherine Bergego Scale (CBS) [9] was developed by Bergego and Azouvi as an assessment tool to identify ADL challenges caused by USN. The reliability and validity of the CBS were excellent, and the sensitivity of the test was superior to that of the paper-and-pencil tests [10]. Right-sided USN in patients with left hemisphere damage was often underdiagnosed because of difficulty assessing USN using paper-and-pencil tests in patients with left hemisphere damage, particularly if they have aphasia and/or severe paresis of the dominant hand. However, the CBS can be used in patients with aphasia and severe dominant hand paresis because it is based on behavioral observations [3, 4]. Using the CBS, Azouvi reported that 77.3% of patients with left hemisphere damage had USN [11]. Recent studies reported that right-sided USN was not rare and was a strong negative predictor of poor rehabilitation outcomes [3, 4]. Furthermore, the scores of paper-and-pencil tests have a tendency to improve when the tests are repeated regardless of the improvement of USN owing to the learning effect [12]. For this reason, CBS is often used to more accurately evaluate the treatment effect of rehabilitation methods for USN [11]. Therefore, behavioral assessments, including CBS, are more appropriate than paper-and-pencil tests to evaluate bilateral USN during the subacute rehabilitation phase.

Chen et al. proposed the Kessler Foundation Neglect Assessment Process (KF-NAP) [13, 14], which is a new scoring method for the CBS based on detailed instructions for observation and scoring. The KF-NAP assessed the perception of left and right space and asymmetrical behaviors by directly observing patients when they explore space with eye and head movements during their daily living activities. Therefore, it can be useful to precisely score CBS even for medical staff unfamiliar with the method of assessment. In addition, it is possible to accurately detect changes in USN in subacute stroke patients during rehabilitation using the KF-NAP.

Our hospital is a rehabilitation hospital in Japan that provides intensive rehabilitation for subacute stroke patients hospitalized within 2 months after onset. In this phase, most patients are in the process of functional recovery, and improvement of USN strongly affects rehabilitation outcomes [4]. Therefore, a precise evaluation of USN is important for proper planning of rehabilitation treatment in patients during this phase. We assessed USN using the CBS since our hospital opened in 2018. We translated the KF-NAP into Japanese and implemented its use in 2019 to score CBS more precisely. The purpose of this study is to evaluate the reliability and validity of our Japanese version of KF-NAP (KF-NAP-J) and to determine whether the introduction of KF-NAP-J has improved the accuracy of neglect assessment in subacute stroke patients during rehabilitation.

## 2. Materials and Methods

### 2.1. Participants and Ethical Considerations

We recruited participants from patients with cerebral stroke who were admitted to Saiseikai Higashi-Kanagawa Rehabilitation Hospital from July 2018 to June 2019, before implementing KF-NAP-J at our hospital, for the CBS group, and from July 2019 to August 2020 for the KF-NAP group. A chief occupational therapist, who did not participate in this study, randomly assigned patients for screening. We included participants admitted within 2 months after the onset of stroke and who could follow the examiner's instructions. We excluded participants whose behavior could not be assessed with the CBS or the KF-NAP-J due to severe aphasia, severe cognitive impairment, severe deafness, or blindness. For the CBS group, we screened 25 stroke patients, and 23 patients fulfilled the inclusion criteria. For the KF-NAP group, of the 39 patients who were screened, 26 fulfilled the inclusion criteria. Four patients were excluded: three had severe aphasia, and one was severely deaf. Therefore, we eventually recruited 23 participants in the CBS group and 22 in the KF-NAP group. We calculated the sample size according to the study by Doros [15]. We assumed the ICC estimate to be 0.8 from previous studies [16, 17], two raters (*k* = 2) assessment, 95% confidence interval (CI), and CI width 0.4; we calculated the minimum sample size as *n* = 20. We determined that 20 or more participants were required.

Experiments were performed according to the Declaration of Helsinki and were approved by the Saiseikai Higashi-Kanawa Rehabilitation Hospital Research Ethics Committee (approval number: HKR0001 and HKR0023). All participants provided written informed consent.

### 2.2. Instrument Translation Process

The KF-NAP was translated into Japanese (forward translation) by a professional life science translator. The comparison and panel discussion led to the primary Japanese version. The translators translated the Japanese form back to English (backward translation), and this was checked and verified by members of the Kessler Foundation who developed the KF-NAP. Finally, the committee comprised two physiatrists and two occupational therapists, who compared the original English form and the obtained form to produce the final Japanese version (KF-NAP-J).

### 2.3. Measurements

The CBS [9] was based on direct observation of the patient's functioning in 10 real-life situations. For each subscale, a 4-point scale was used, ranging from 0 (no neglect) to 3 (severe neglect). The total score was calculated (range: 0–30). The CBS also included self-assessment to measure patients' awareness of the neglect-related activity of daily life. A parallel form of the CBS was used as a questionnaire with the same 10 subscales mentioned above. For the subscales that could not be measured, the average score of the measured items was entered. In this study, we used the observer's assessment as a neglect assessment.

The KF-NAP [13, 14] was a new scoring method for the CBS based on detailed instructions for observation and scoring. It could more precisely assess the perception of both space and asymmetrical behaviors by directly observing patients when they explored space with eye and head movements during their daily living activities. It included the 10 criteria of the KF-NAP: limb awareness, personal belongings, dressing, grooming, gaze orientation, auditory attention, navigation, collisions, meals, and cleaning after meals. For each item, a 4-point scale was used, ranging from 0 (no neglect) to 3 (severe neglect), the same as the CBS.

The BIT is a standard test used to assess USN and consists of conventional and behavioral tests. The conventional test consists of six subscales (line crossing, letter cancellation, star cancellation, figure and shape copying, line bisection, and representational drawing), with a score ranging between 0 and 146; a higher score indicates a better spatial awareness. In the original version, the cut-off score is 129 [7], whereas it is 131 in the Japanese version [18]. We defined participants whose score was ≤131 as having USN. In this study, we used the conventional BIT for the participants in the KF-NAP group.

The functional independence measure (FIM), which was developed to ensure uniformity in assessing the ADLs, includes motor and cognitive subscales and is subdivided into 18 items [19]. For each subscale, a 7-point scale was used, ranging from 1 (total assistance) to 7 (complete independence), and the total score ranged from 18 to 126; higher scores indicate greater independence in the ADLs. FIM included the motor subscales (13 items: eating, grooming, bathing, dressing the upper body, dressing the lower body, toileting, bladder control, bowel control, transfer to bed/chair/wheelchair, toilet transfer, transfer to tub/shower, walking or wheelchair use, and stairs) and the cognitive subscales (5 items: comprehension, expression, social interaction, problem-solving, and memory). This score is assessed by a physiatrist or a nurse who treated the participants and was trained in FIM scoring.

The Mini-Mental State Examination (MMSE) is a simple examination used to evaluate the decline in cognitive function (faculty of orientation, memory, calculating ability, language ability, and constructional ability) and is conducted using a verbal questionnaire [20]. It is composed of 11 items, with the score ranging between 0 and 30. In this study, we used the Japanese version of the MMSE (MMSE-J) that is officially licensed by Psychological Assessment Resources Inc. (PAR) [21, 22]. The validity and reliability of the MMSE-J were well documented [22].

### 2.4. Procedure

Two OTs (who had not participated in translating the KF-NAP) assessed participants using the CBS (CBS group). Two other OTs who were members of the KF-NAP-J translation team and trained in KF-NAP assessment assessed participants using the KF-NAP-J (KF-NAP group). The raters of both the CBS and the KF-NAP-J possessed similar clinical experience and skills. The ADLs in both groups were assessed using the FIM. The USN of the KF-NAP group was assessed using the conventional BIT.

### 2.5. Analysis

#### 2.5.1. Baseline Characteristics

The two groups were compared by performing the unpaired *t*-test (age, disease duration, Mini-Mental State Examination (MMSE), and FIM) and chi-squared test (sex, lesion, and stroke subtype). The level of statistical significance for the variables was set at 0.05.

#### 2.5.2. Reliability

Interrater reliability was calculated employing intraclass correlation coefficients (ICC) for total scores and weighted kappa coefficients for each subscale. Internal consistency was calculated using Cronbach's alpha. The level of statistical significance for the variables was set at 0.05.

#### 2.5.3. Validity

The KF-NAP was evaluated for convergence and discriminative validity of Rater 1. Convergence validity was obtained using the Spearman correlation coefficient of the KF-NAP against conventional BIT and FIM. The level of statistical significance for the variables was set at 0.05.

#### 2.5.4. USN Detection Rate

In the KF-NAP group, we calculated and compared the percentage of patients detected with USN symptoms by KF-NAP and the percentage of patients who were below the cut-off point with the BIT conventional test by Rater 1. In addition, we calculated the detection rates of the injured side in the participants assessed using the KF-NAP and BIT.

Data analysis was performed using IBM SPSS Statistics for Mac, Version 23 (Armonk, NY, USA).

## 3. Results

### 3.1. Participants

The demographic data of all participants are presented in Table 1. There was no significant difference between the two groups with respect to age, sex, type of stroke, disease duration, and FIM (*P* > 0.05). The CBS group had more patients with left hemisphere damage and lower MMSE scores than the KF-NAP group. Four participants in the CBS group could not participate in MMSE assessment due to motor aphasia. However, CBS assessment in these participants could be performed because they could understand and follow the instructions.

### 3.2. Reliability

The ICC and Cronbach's alpha of the two groups are shown in Table 2. Both the CBS and KF-NAP exhibited excellent interrater reliability and internal consistency. However, it appears that the KF-NAP exhibited slightly better reliability than the CBS.

The weighted kappa coefficients of each subscale of the KF-NAP and CBS are shown in Table 3. In the CBS, 3 of 10 subscales (limb awareness, dressing, and meals) did not exhibit significant agreement between the two raters, whereas the other subscales were in fair to substantial agreement. In the KF-NAP, weighted kappa coefficients could not be calculated for auditory attention and navigation because one rater assigned the same score (zero) on the subscales for all the participants. In such a case, the weighted kappa cannot be calculated due to the calculation methods used [23]. However, the weighted kappa for all eight subscales was in significant agreement, and two subscales (gaze orientation and cleaning after meals) were almost in perfect agreement.

The order of the CBS was changed to correspond with that of KF-NAP.

### 3.3. Validity

The correlation between the KF-NAP and conventional BIT is shown in Table 4. Four of six subscales of BIT were significantly correlated with KF-NAP, and the total score also tended to be correlated with KF-NAP.

**Figure 1:**
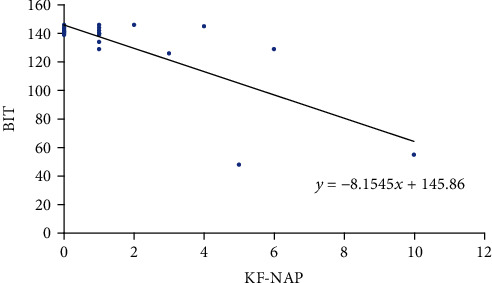
Correlation between KF-NAP and BIT total.

**Figure 2:**
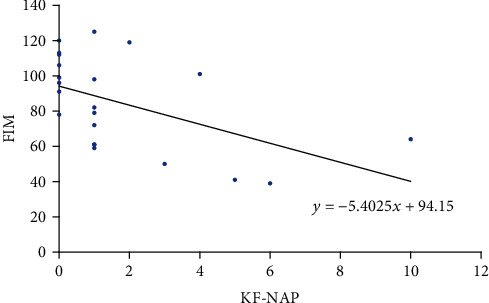
Correlation between KF-NAP and FIM total.

**Figure 3:**
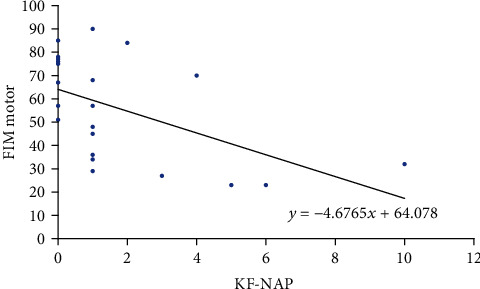
Correlation between KF-NAP and FIM-motor.

The correlation between the KF-NAP and FIM is shown in Table 4 and Figures 1–3. Total FIM and motor FIM were correlated with KF-NAP. However, cognitive FIM was not correlated with KF-NAP.

The correlation between the KF-NAP and FIM is shown in Table 4 and Figures 1 and 3. Total FIM and motor FIM were correlated with KF-NAP. However, cognitive FIM was not correlated with KF-NAP.

### 3.4. USN Detection Rate

The USN detection rates of KF-NAP and BIT in the KF-NAP group are shown in Table 5. In the KF-NAP group, KF-NAP could detect more patients with USN than BIT, particularly in patients with left hemisphere damage.

## 4. Discussion

We evaluated USN using the KF-NAP in subacute stroke patients during rehabilitation in Japan. In the Japanese healthcare system, stroke patients are transferred to a rehabilitation hospital within 60 days, and the time between stroke onset and admission to rehabilitation hospitals is generally 3 to 5 weeks. Subsequently, patients receive intensive rehabilitation treatment for several months.

In this study, both interrater reliability and internal consistency were slightly better in the KF-NAP group than in the CBS group. Each subscale was generally in better agreement in the KF-NAP group than in the CBS group. Previous studies reported that both CBS [9, 10] and KF-NAP [8, 16] displayed excellent reliability. However, to the best of our knowledge, comparisons of interrater reliability between CBS and KF-NAP were not reported. Since KF-NAP was developed for scoring CBS more easily and precisely [8, 11], we hypothesized that the introduction of KF-NAP would improve the reliability of CBS. First, we assessed USN using the CBS and subsequently assessed USN using the KF-NAP in the different patient groups at the same hospital. These results support the hypothesis.

In this study, weighted kappa coefficients of two subscales (auditory attention and navigation) could not be calculated because one rater assigned the same score (zero) on the subscales for all the participants. However, the other rater also scored zero for all except two participants on the auditory attention (scored 1) and except for one (scored 1) on the navigation. Therefore, even these two subscales were in good agreement between the raters. Although most subscales of KF-NAP were in moderate or better agreement, only the personal belonging subscale remained fair even with KF-NAP, possibly due to the assessment method and the space constraints in the Japanese hospital room setting. We assessed the personal belonging subscale from 3 to 6 based on the patients' daily use of personal belongings placed in the room and assessed their behavior. It is difficult to evenly position personal belongings around a patient on the left and right because of limited personal space in the hospital rooms in Japan. Therefore, it is challenging to appropriately arrange the room for the assessment. The interrater reliability of the subscales was less consistent than in previous reports both in the KF-NAP and the CBS. The possible reasons are as follows: (1) the participants were in the subacute intensive rehabilitation ward, and most of the patients experience improvement in their function and abilities day by day, and the symptoms of USN fluctuated. Therefore, we expect that the scores would be significantly different between raters compared with those of patients during the acute phase, as reported in previous studies. (2) Most participants with USN had mild to moderate symptoms. Potentially, in these patients, USN symptoms would have been impacted by intensive rehabilitation more rapidly than in patients with more severe USN, and (3) the raters were not adequately trained, which could be improved by thorough education.

In our study, KF-NAP-J was correlated with the line cancellation test, star cancellation test, and figure and shape copying of BIT. Azouvi et al. [10] reported significant correlations between the CBS and three paper-and-pencil tests, including the Bells test, the copying test, and the sentence reading test in patients with USN. Kim et al. [17] demonstrated that KF-NAP was correlated with the Albert's test and letter cancellation test. Our results suggest that KF-NAP-J could reflect behavioral changes related to USN symptoms such as visual exploration and spatial representation that are assessed by paper-and-pencil tests, as well as the original version of CBS and KF-NAP. However, the correlation coefficients were relatively small in our study. In this study, only 22.7% of patients were diagnosed as USN by BIT, while 63.6% were diagnosed by KF-NAP-J. This could be due to the difference in detection rates between BIT and KF-NAP.

KF-NAP-J was also correlated with motor and total FIM but not with cognitive FIM. Chen et al. [13] showed that the KF-NAP had significant correlations with FIM and the Barthel Index. The subscales of CBS and KF-NAP assess the difficulties in daily life situations due to USN. Moreover, USN affects motor FIM more than cognitive FIM [6]. Our study is therefore consistent with previous studies.

The USN detection rate of the KF-NAP was higher than that of the BIT. Subacute patients receiving rehabilitation treatment may learn to compensate for USN symptoms in the visual search task. In addition, paper-and-pencil tests tend to improve when the tests are repeated regardless of improvement of USN [12]. Therefore, behavioral tests like the KF-NAP were more accurate at detecting USN for these patients [11].

In patients with left hemisphere damage, the USN detection rate of KF-NAP was considerably higher than that of BIT. No USN patients were detected using BIT, whereas 60% were diagnosed with USN by KF-NAP. It is difficult to evaluate USN using paper-and-pencil tests if the patients have aphasia and/or severe dominant hand paresis. However, right-sided USN of patients with left hemisphere damage equally predicts poor functional outcomes as left-sided USN [3, 4]. Therefore, behavioral testing such as KF-NAP is particularly useful for patients with left hemisphere damage.

USN is a strong negative predictor of poor rehabilitation outcomes [2, 6, 24], and recovery of USN affects the achievement of ADLs [6, 25–27] and discharge destination [4] after rehabilitation. Therefore, it is very important to accurately assess USN for subacute stroke patients during rehabilitation. KF-NAP-J exhibited good reliability and validity and may be useful for predicting rehabilitation outcomes and planning appropriate rehabilitation treatment.

One of the main limitations of this study is the small sample size. There were only 22 participants in the KF-NAP group and 25 participants in the CBS group. Therefore, we could not match the lesion side and distribution of severity between the two groups. Future studies with a large number and a wide spectrum of participants are required to ascertain the reliability and validity of the method. In addition, we assigned the participants and the raters to either the CBS group or the KF-NAP group. Ideally, to effectively compare the reliability between the CBS and KF-NAP is to assess the same participants by two KF-NAP trained raters and two other raters trained with the original CBS but not KF-NAP trained. However, it is also difficult to retain blindness and independence among raters if many raters assess the same participants in a small hospital. Therefore, we initially separated the raters for the two groups, and two raters assessed participants in the CBS group, whereas the other two raters were involved in translating and KF-NAP training. Owing to these limitations in the study design, we cannot confirm the validity and reliability based on the results of this study only. Despite these limitations, the investigators believe that the KF-NAP-J is useful for assessing CBS in stroke patients undergoing subacute rehabilitation.

## 5. Conclusions

In this study, the KF-NAP exhibited good interrater reliability and correlated with the subscales of BIT and FIM, which represent USN and ADLs in subacute stroke patients. Unilateral spatial neglect strongly impacts rehabilitation training and its outcomes, therefore requiring accurate assessment of USN. In addition, behavioral testing is even more important in patients with left hemisphere damage because it facilitates assessment even if the patients experience aphasia and paralysis of the right hand. These results suggest that KF-NAP and CBS are useful in assessing USN in subacute stroke patients.

## Figures and Tables

**Table 1:** Characteristics of the participants.

		KF-NAP group (*n* = 22)	CBS group (*n* = 23)	*P*
Age		65.4 ± 13.5	64.4 ± 12.2	0.941
Sex (male/female)		17/5	17/6	0.66
Disease duration (day)		80.7 ± 64.5	99.6 ± 36.9	0.405
Lesion (right/left/bilateral)		16/5/1	9/14/0	0.045^∗^
Stroke subtype Ischemic stroke/hemorrhagic stroke		11/11	15/8	0.378
MMSE		26.1 ± 3.7	21.8 ± 7.2^∗∗^	0.026^∗^
FIM total		84.8 ± 26.3	89.9 ± 23.8	0.310
Motor		56.0 ± 22.2	62.9 ± 19.4	0.158
Cognitive		28.8 ± 5.6	27.0 ± 6.3	0.381
BIT total		131.8 ± 26.6		
Line cancellation		34.8 ± 4.8		
Letter cancellation		34.7 ± 8.6		
Star cancellation		49.5 ± 10.9		
Figure and shape copying		2.5 ± 1.4		
Line bisection		7.9 ± 2.5		
Representational drawing		2.3 ± 1.1		
KF-NAP total score	(Rater 1)	1.73 ± 2.51		
	(Rater 2)	1.91 ± 2.29		
CBS total score	(Rater 3)		2.39 ± 4.00	
	(Rater 4)		2.39 ± 4.52	

^∗∗^MMSE could not be assessed in 4 CBS participants due to motor aphasia. Comparison between the two groups was performed using the unpaired *t*-test (age, disease duration, MMSE, FIM) and chi-squared test (sex, lesion, stroke subtype). The level of statistical significance for the variables was set at *P* < 0.05.

**Table 2:** Interrater reliability of KF-NAP and CBS.

	KF-NAP group	*P*	CBS group	*P*
ICC	0.921	<0.001	0.852	<0.001
Cronbach's alpha	0.969		0.904	

**Table 3:** Interrater reliability of each subscale of KF-NAP and CBS.

	KF-NAP group		CBS group	
Subscale	Weighted kappa	*P*	Weighted kappa	*P*
Gaze orientation	0.89	<0.001	0.372	0.016
Limb awareness	0.56	<0.001	0.27	0.087
Auditory attention	—		0.535	0.001
Personal belongings	0.353	0.0035	0.297	0.013
Dressing	0.522	<0.001	0.361	0.051
Grooming	0.66	<0.001	0.372	0.016
Navigation	—		0.61	<0.01
Collisions	0.654	<0.001	0.618	<0.001
Meals	0.645	0.001	0.33	0.104
Cleaning after meals	1	<0.001	0.303	0.008

Weighted kappa coefficients of “auditory attention” and “navigation” in KF-NAP could not be calculated because one rater assigned the same score on the subscale to all the participants.

**Table 4:** Correlation between KF-NAP and other assessments.

Spearman	*r*	*P*	*R ^2^*
BIT total	-0.405	0.062	0.592
Line cancellation	-0.495	0.019^∗^	0.204
Letter cancellation	-0.278	0.210	0.634
Star cancellation	-0.437	0.042^∗^	0.587
Figure and shape copying	-0.43	0.046^∗^	0.288
Line bisection	-0.282	0.204	0.528
Representational drawing	-0.445	0.038^∗^	0.325
FIM total	-0.521	0.013^∗^	0.266
Motor	-0.565	0.006^∗^	0.280
Cognition	-0.334	0.129	0.107

BIT: Behavioral Inattention Test; FIM: Functional Independent Measure. ^∗^Significant correlation (Spearman correlation coefficient; *P* < 0.05). *R*^2^: least square of regression lines.

**Table 5:** USN detection rates measured in the KF-NAP group.

	Total %	Right lesion %	Left lesion %
KF-NAP (*n* = 22)	63.6	68.8	60.0
BIT (*n* = 22)	22.7	31.2	0

## Data Availability

Data are available upon reasonable request to the corresponding author.
